# Role of mitophagy in ocular neurodegeneration

**DOI:** 10.3389/fnins.2023.1299552

**Published:** 2023-10-27

**Authors:** Calvin D. Brooks, Bindu Kodati, Dorota L. Stankowska, Raghu R. Krishnamoorthy

**Affiliations:** ^1^Department of Pharmacology and Neuroscience, University of North Texas Health Science Center, Fort Worth, TX, United States; ^2^North Texas Eye Research Institute, University of North Texas Health Science Center, Fort Worth, TX, United States

**Keywords:** mitochondria, mitophagy, glaucoma, age-related macular degeneration, diabetic retinopathy, neurodegeneration

## Abstract

Neurons in the central nervous system are among the most metabolically active cells in the body, characterized by high oxygen consumption utilizing glucose both aerobically and anaerobically. Neurons have an abundance of mitochondria which generate adequate ATP to keep up with the high metabolic demand. One consequence of the oxidative phosphorylation mechanism of ATP synthesis, is the generation of reactive oxygen species which produces cellular injury as well as damage to mitochondria. Mitochondria respond to injury by fusion which serves to ameliorate the damage through genetic complementation. Mitochondria also undergo fission to meet an increased energy demand. Loss of mitochondria is also compensated by increased biogenesis to generate new mitochondria. Damaged mitochondria are removed by mitophagy, an autophagic process, in which damaged mitochondria are surrounded by a membrane to form an autophagosome which ultimately fuses with the lysosome resulting in degradation of faulty mitochondria. Dysregulation of mitophagy has been reported in several central nervous system disorders, including, Alzheimer’s disease and Parkinson’s disease. Recent studies point to aberrant mitophagy in ocular neurodegenerative disorders which could be an important contributor to the disease etiology/pathology. This review article highlights some of the recent findings that point to dysregulation of mitophagy and it’s underlying mechanisms in ocular neurodegenerative diseases, including, glaucoma, age-related macular degeneration and diabetic retinopathy.

## Introduction

### Mitochondria and bioenergetics

Mitochondria are the power grid of eukaryotic cells producing ATP, the primary energy currency of the cell, which power various biosynthetic and metabolic activities in the cell. Within the mitochondria, the inner membrane is thrown into multiple folds called cristae which greatly increase its surface area and enable the accumulation of several 1,000 copies of components of the electron transport chain (complex I to complex IV). The oxidation of metabolic fuels such as glucose and fatty acid is coupled to the reduction of NAD^+^ and FAD to NADH + H^+^ and FADH_2_, respectively. High-energy electrons extracted from these compounds travel down the electron transport chain along a sequence of electron carriers with a decrease in free energy. During this electron transport, the electron carriers alternate between reduced (as they accept electrons) and oxidized state (as they donate electrons), ultimately culminating in the transfer of the electrons to molecular oxygen, which is the final acceptor of electrons in the electron transport chain. The downhill transport of electrons along the electron transport chain is coupled to the uphill pumping of protons across the inner mitochondrial membrane into the intermembrane space, creating a proton gradient across the inner membrane. The enzyme ATP synthase then harnesses the energy inherent in the electrochemical proton gradient across the inner mitochondrial membrane to generate ATP through chemiosmotic coupling.

### Reactive oxygen species

This exquisite mechanism of oxidative metabolism of glucose and fatty acids, followed by oxidative phosphorylation, is not without perils for the cell. Electron leaks could occur from the electron transport chain, leading to a partial reduction of molecular oxygen, producing superoxide radical, which could be reduced to form hydrogen peroxide. A superoxide molecule could also react with a molecule of hydrogen peroxide molecule to generate hydroxyl radicals by the following net reaction: ^•^O_2_^−^ + H_2_O_2_ → ^•^OH + OH^−^ + O_2_. Superoxides could react nitric oxide to produce the highly reactive peroxynitrite which could have both beneficial and detrimental effects. For instance, neutrophils and macrophages generate peroxynitrite and utilize it for a host-defense response to kill bacterial invading bacteria. However, production of peroxynitrite could be a contributor to ischemia–reperfusion injury in various tissues such as the myocardium and the retina (Vinten-Johansen, 2000; Canto et al., 2019). Additionally, hydroxyl radicals could be formed from hydrogen peroxide by the Fenton reaction through the reaction of hydrogen peroxides and metal ions, including Fe^2+^ and Cu^+^ and these radicals are extremely harmful to cells by their ability to damage membranes, sugar residues, and DNA. While many of these reactive oxygen species are generated during oxidative metabolism in a cell, they are kept in check by various antioxidants, including ascorbic acid, N-acetyl cysteine, and glutathione, present at high cell concentrations in the cell. An excess reactive oxygen species could tilt the redox balance and result in oxidative stress, contributing to various pathologies.

While oxidative phosphorylation is a key function of all cells in the body of an aerobic organism, some very metabolically active tissues, including the brain, liver, muscle, and heart, have a greater propensity to generate ROS. CNS neurons are among the most metabolically active tissues in the body, characterized by high oxygen consumption and generation of ATP required for axonal transport and propagation of action potential, in addition to various metabolic and biosynthetic activities of the cell. It is estimated that while the brain weighs 5% of the total body weight, it accounts for 20% of total oxygen consumption. The retina and optic nerve are specialized regions of the CNS and are also among the most oxygenated and metabolically active tissues in the body; hence, mitochondria play a vital role in the RGCs to power crucial cell activities, including anterograde and retrograde axonal transport and saltatory conduction through the axons of the optic nerve (Calkins, 2012; Inman and Harun-Or-Rashid, 2017). When ROS increase beyond the capacity of cellular antioxidants to contain them, they damage cellular components, which could ultimately manifest in neurodegenerative effects. Several neurodegenerative diseases, including, Alzheimer’s disease, Parkinson’s disease, and age-related macular degeneration, involve mitochondrial dysfunction, particularly a decline in mitochondrial bioenergetics leading to neurodegeneration (Wang et al., 2020; Yang et al., 2023). ROS could also be responsible for propagating neuronal injury as a secondary effect following traumatic injury to the brain and eyes (Ryan et al., 2023). Hondur et al. (2017) reported a significant increase oxidative stress related biomarkers (protein carbonyls and advanced glycation end products) in both aqueous humor and blood samples from glaucoma patients, compared to healthy subjects (Hondur et al., 2017). Mitochondrial mutations contribute to ocular neurodegenerative diseases, including Leber’s Hereditary Optic Neuropathy (LHON) and Neurogenic weakness, Ataxia, and Retinitis Pigmentosa (NARP; Schrier and Falk, 2011). Three mutations in the electron transport chain’s complex I (NADH dehydrogenase) account for 80% of patients with Leber’s Hereditary Optic Neuropathy (Schrier and Falk, 2011). The retina and optic nerve depend highly on oxidative phosphorylation as a mechanism of energy metabolism to generate ATP, which could account for optic nerve degeneration as the primary manifestation of the Leber’s Hereditary Optic Neuropathy rather than resulting in a systemic disease.

### Mitochondrial fusion and fission

Mitochondria respond to DNA damage by fusion, enabling genetic complementation to ameliorate the genetic defects generated by the injury. Mitochondria also undergo fission to generate additional mitochondria to meet higher energy demands of the cells or for the distribution of mitochondria to daughter cells during cell division. By constant fusion and fission, mitochondria form an interconnected network within eukaryotic cells. Using a computational model of mitochondrial networks, Zamponi et al. (2018) found that structural parameters of healthy mitochondria lay in between the extremes of highly fragmented and complete fusion networks. Impaired mitochondrial fusion and fission alter in the mitochondrial network, causing loss of mitochondrial DNA, producing bioenergetic compromise in mammalian cells (Ju et al., 2023). These changes can be brought about by alteration in the expression of mitofusion proteins (MFN1/2 and OPA1) involved in mitochondrial fusion as well as by the dynamin-like protein Drp-1, which participates in mitochondrial fission. For instance, an increase in mitochondrial fission and a decrease in fusion are associated with neurodegeneration in animal models of glaucoma (Ju et al., 2023). Excessive fission produces immature mitochondria with poor cristae formation and decreased capacity for ATP generation. Increased mitochondrial fragmentation mediated by Drp-1 and its association with Aβ and hyperphosphorylated Tau has been demonstrated in Alzheimer’s and Parkinson’s disease (Lawrence et al., 2022).

### Mitophagy

Mitochondria can undergo damage by various mechanisms, including overproduction of ROS, elevated Ca^2+^, increased fission, and decreased fusion. These cellular insults and changes in mitochondrial dynamics could result in an accumulation of damaged/dysfunctional mitochondria in the cell which could perpetuate the damage by production of additional ROS. One of the quality control mechanisms by which damaged mitochondria are eliminated is mitophagy. In this process, damaged mitochondria are enveloped by a membrane derived primarily from the endoplasmic reticulum to form a double membraned structure called phagophore. The elongation of the phagophore membrane to form the autophagosome involves ubiquitination reactions catalyzed by the ATG family of proteins. Initially the ubiquitin-like molecule Atg12 is conjugated to Atg5 to form the Atg12-Atg5 complex, which interacts non-covalently with Atg16L1. The subsequent ubiquitin-like reaction involves conjugating the Atg8 family, which includes members of three subfamilies: LC-3, GABARAP, and GATE16. Microtubule-associated protein 1 light chain 3 (MAP-LC3/Atg8/LC3) is the best-characterized member of the Atg8 family, which is conjugated with phosphatidyl ethanolamine by Atg7 (E1-like) and Atg3 (E2-like) producing an autophagosome-associated LC3-II. Thus, LC3-positive vesicles, can be considered to be completed autophagosomes (Rubinsztein et al., 2012).

**Figure 1:**
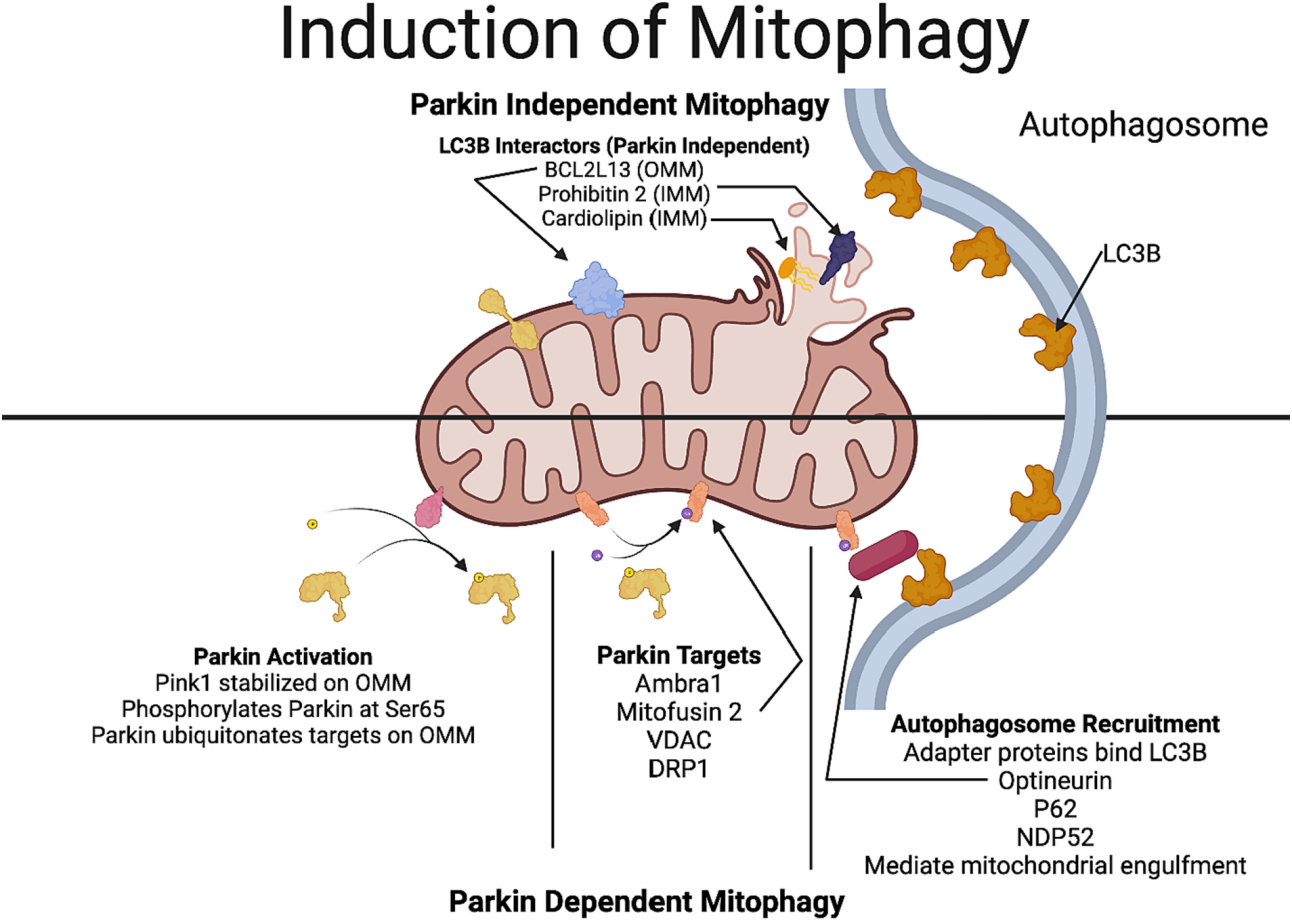
Mechanisms involved in Parkin-dependent and Parkin-independent mitophagy. In Parkin-dependent mitophagy, due to the decline in the mitochondrial potential, PINK1 gets stabilized in the outer mitochondrial membrane. PINK1 recruits Parkin (a ubiquitin ligase), which ubiquitinates several outer mitochondrial proteins including VDAC, mitofusin and DRP1. Optineurin and NDP52, which have a LC3-interacting region that act as preferential receptors for the ubiquitinated proteins to connect with the LC3 protein in the autophagosome membrane, thereby facilitating the engulfment of the mitochondria. In Parkin-independent pathway, proteins in the outer mitochondrial membrane, including, BNIP3, NIX/BNIP3L, FUNDC1, FKBP8, and Bcl2L13 which have a LC3-interacting region, directly interact with the autophagosome promoting the engulfment of the mitochondria by the autophagosome. Additionally, rupture of the outer mitochondrial membrane reveals more LC3-interactors such as prohibitin 2 and cardiolipin. Figure created using Biorender.

The majority of work on mitophagy revolves around the ubiquitin ligase Parkin and the kinase PINK1 (PTEN-induced putative kinase 1). In healthy mitochondria, having an intact mitochondrial transmembrane potential, PINK1 is imported to the inner mitochondrial membrane, where it is cleaved by a protease called rhomboid protease presenilin-associated-rhomboid-like (PARL) and subsequently degraded in the proteasome. Following oxidative stress and other cellular stressors, such as the elevation of intracellular Ca^2+^, there is a collapse of the mitochondrial potential; hence, PINK1 can no longer be imported into the inner mitochondrial membrane, hence it is stabilized in the outer mitochondrial membrane. PINK undergoes autophosphorylation and phosphorylates ubiquitin and Parkin, thereby recruiting Parkin to the outer mitochondrial membrane and activating it. The activated Parkin ubiquitinates numerous mitochondrial proteins, including mitofusin 2 (Mfn2), voltage-dependent anion-selective channel protein (VDAC), and dynamin-1-like protein (DRP-1; Figure 1). These ubiquitinated proteins in the outer mitochondrial membrane, recruit several autophagy receptors, namely, optineurin (OPTN), calcium-binding and coiled-coil domain 2 (NDP52) and sequestosome 1 (p62), tax 1 binding protein 1 (TAX1BP1) and next to BRCA1 gene1 protein (NBR1). Among these autophagy receptors, OPTN and NDP52 are usually the preferred adaptors, having an LC3-interacting region to interact with the LC3 protein in the autophagosome membrane. This interaction between the autophagy receptor and the autophagosome brings it closer to engulfing damaged mitochondria in the autophagosome (Figure 1). Subsequently, the autophagosome undergoes fusion with the lysosome to facilitate the entry of the damaged mitochondria into the lysosome and its degradation. Regulators of PINK1 localization are potential targets for therapeutic approaches to modulate mitophagy, Chief among them is ATPase family AAA domain containing 3A (ATAD3A), a protein that regulates PINK1 importation and degradation, and autophagy and beclin 1 regulator 1 (AMBRA1) which can inhibit the action of ATAD3A and promote PINK1 stabilization on the outer mitochondrial membrane (Di Rienzo et al., 2022).

In receptor-mediated mitophagy, proteins in the outer mitochondrial membrane including, BNIP3, NIX/BNIP3L, FUNDC1, FKBP8, and Bcl2L13 that contain an LC3-interacting domain directly bind to LC3, allowing the direct recruitment of the phagophore to damaged mitochondria in a parkin-independent manner (Villa et al., 2018). Additionally, rupture of the outer mitochondrial membrane can reveal proteins such as prohibitin 2 and specialized phospholipids like cardiolipin on the inner mitochondrial membrane that can interact with LC3B to induce mitophagy (Villa et al., 2018). Parkin-independent mitophagy is underexplored in the context of neurodegenerative diseases.

### Mitochondrial biogenesis

Loss of mitochondria could be compensated by the increased mitochondrial biogenesis characterized by growth and division of existing mitochondria. Mitochondrial biogenesis occurs in response to an increased energy demand as well as developmental signals and various environmental stresses. In humans, only 1% of mitochondrial proteins are encoded by the mitochondrial genome; the remaining 99% of mitochondrial proteins are encoded in the nuclear genome. While the majority of the mitochondrial proteins are translated in the cytoplasm and imported into the mitochondria, some the mitochondrial proteins are synthesized within the mitochondria utilizing mitochondrial ribosomes, the mechanistic details are still being elucidated (Khawaja et al., 2023). Many of the genes and proteins involved in mitochondrial biogenesis have been identified, however, the regulation of mitochondrial biogenesis and signaling mechanisms governing it have not been completely elucidated (Popov, 2020). Transcription of mitochondrial DNA is activated by the PPARγ coactivator-1 (PGC) family which includes PGC1α, PGC1β and PRC which are involved in homeostatic mechanisms governing glucose, lipid, and energy metabolism and likely contributors to the pathogenesis of obesity, diabetes, neurodegeneration, and cardiomyopathy (Lin et al., 2005). Mitochondrial biogenesis is regulated by the transcription factor peroxisome-proliferator-activated receptor gamma coactivator-1α (PGC-1α) a master regulator or mitochondrial biogenesis (Popov, 2020). PGC-1α regulates the nuclear transcription factor nuclear respiratory factor-1 (NRF-1) and NRF-2 which in turn activate transcription factor A, mitochondrial (TFAM). Together, these factors regulate mitochondrial replication and repair. There is a fine coordination between the process of mitophagy (to remove damaged mitochondria) and mitochondrial biogenesis (to generate new mitochondria) in order to maintain homeostasis and a healthy mitochondrial network (Popov, 2020).

**Figure 2:**
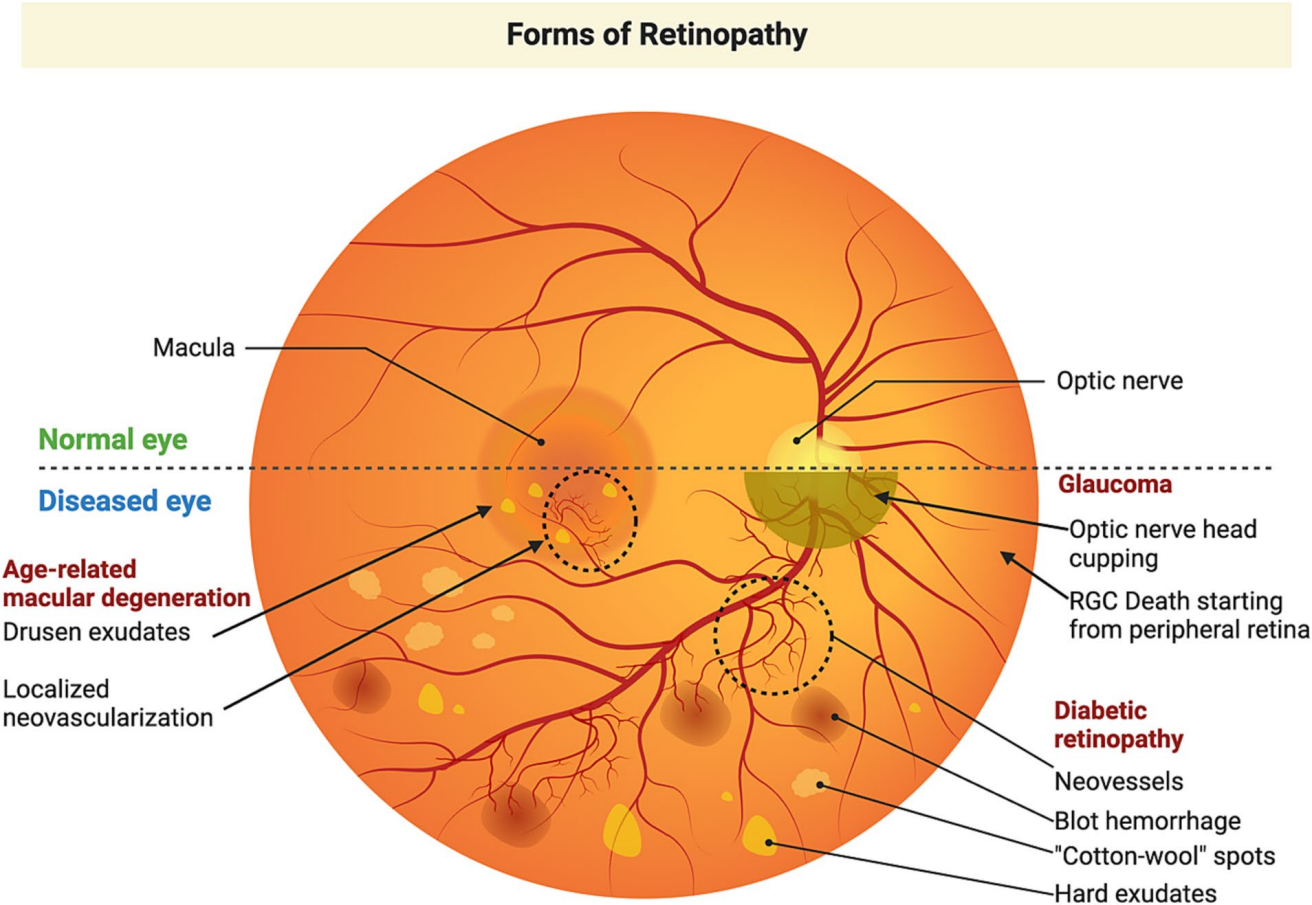
Etiology of retinal neurodegenerative diseases. Glaucoma is an optic neuropathy commonly associated with an elevation of intraocular pressure and characterized by degeneration of the optic nerve, cupping of the optic disk, and retinal ganglion cell loss, leading to peripheral vision loss, which could ultimately lead to complete loss of vision. Age-related macular degeneration primarily affects the macular region and is characterized by the accumulation of drusen (lipofuscin-like material) posterior to the RPE. As the disease progresses, accumulation of larger drusen disrupts the blood-retinal barrier. Subsequently, there is growth of new blood vessels penetrating the RPE. These newly formed blood vessels are fragile and get ruptured and leak their contents producing a devastating loss of vison. Diabetic retinopathy is a secondary manifestation of diabetes, which starts as mild non-proliferative diabetic retinopathy, characterized by microaneurysms in retinal blood vessels. These lead to compromised circulation and hypoxia in parts of the retina which triggers the formation of new blood vessels that leak and produce rapid vision loss. Figure created using Biorender.

The involvement of mitochondrial dynamics and mitophagy has garnered increasing attention in the past decade, particularly in neurodegenerative diseases. This review will focus on mitophagy in three ocular neurodegenerative diseases: glaucoma, age-related macular degeneration and diabetic retinopathy (Figure 2). Insights gained from these ocular neurodegenerative diseases could point to similar mechanisms by which a decline in mitophagy may contribute to neurodegeneration in other brain neurodegenerative diseases.

## Mitophagy in glaucoma

Glaucoma is an age-related neurodegenerative disease of the eye, and is a leading cause of blindness in the developed world, with 3 million patients in the United States and nearly 80 million patients worldwide (Boland and Quigley, 2007; Weinreb et al., 2016). The cause of vision loss in glaucoma is the degeneration of the optic nerve and loss of retinal ganglion cells (McMonnies, 2018). Mitochondrial dysfunction has been shown to have a prominent role in the death of RGCs in glaucoma (Abu-Amero et al., 2006; Nguyen et al., 2011; Jassim et al., 2021; Tribble et al., 2021). As early as 2006, Abu-Amero et al. had shown that patients with an inherited form of glaucoma also had novel mutations in mitochondrial DNA caused by oxidative stress (Abu-Amero et al., 2006). Several markers of oxidative stress have been found in the aqueous humor as well as plasma samples from glaucoma patients (Pinazo-Duran et al., 2013).

Sacca and Izzotti (2008) suggested that oxidative stress mediated degenerative changes in the trabecular meshwork as well as the retinal ganglion cells could be contributors to glaucoma pathology.

Oxidative stress could have damaging effects on the mitochondria and produce a decline in mitochondrial potential. Studies in several animal models of glaucoma have consistently shown that elevations in intraocular pressure have increased mitochondrial fission, increased expression of pro-apoptotic peptides, disrupted small molecule metabolism, and reduced respiratory capacity (Nguyen et al., 2011; Williams et al., 2017; Jassim et al., 2021; Tribble et al., 2021). As discussed previously, damaged mitochondria are typically cleared through mitophagy; however, studies on mitophagy in glaucoma are limited.

While mitochondria have been known to contribute to glaucoma for several years, examining mitophagy in glaucoma has gained considerable attention recently. In one of the earliest findings, Davis et al. (2014) reported that in the optic nerve head region in DBA/2 J mice, axonal mitochondria are not degraded within the axons by traditional mitophagy, but rather shed from them and taken up by the resident astrocytes through a process the authors termed as transcellular degradation of mitochondria, or transmitophagy. Coughlin et al. (2015) were among the first to attempt to study mitophagy in glaucoma. Using the DBA/2 J mouse model of glaucoma, they examined the optic nerves of these mice using transmission electron microscopy (TEM; Coughlin et al., 2015). They found that glaucomatous mice had more mitochondria, but mitochondria were often malformed and lacking cristae, signs that the mitochondria were not healthy (Coughlin et al., 2015). More autophagosomes were visible under TEM, and this result was confirmed using western blotting for LC3B (Coughlin et al., 2015). However, there was no evidence of an increase in mitophagy (Coughlin et al., 2015). The researchers suggest an increase in damaged mitochondria, with no change in the levels of Pink1 and Parkin, two proteins involved in mitophagy, are indicative of a decline in mitophagy (Coughlin et al., 2015). However, it is hard to gauge the activity of an active process such as mitophagy, where the endpoint is the degradation of the target, if the process is successful, there is no evidence left behind. It could be argued that mitophagy is increased, just not to the degree necessary to keep up with the continued mitochondrial damage.

Zeng et al. (2020) found similar results when using the dexamethasone-induced model of ocular hypertension; western blot analysis showed an increase in autophagosome formation (as measured by LC3B), while P62, a protein ubiquitinated by Parkin to signal mitophagy, was decreased. Obanina et al. (2022) examined mitophagy markers in cross-sections of human eyes with and without glaucoma, and found that autophagy markers were more widespread throughout the retina in glaucoma patients; however, P62 was markedly reduced. Hass and Barnstable found that knocking out Mitochondrial Uncoupling Protein 2 (UCP2) in astrocytes, a glial cell that interacts with RGCs, restored respiratory capacity and increased mitophagy (as measured by LC3B and Tom20 colocalization) inside astrocytes and promoted RGC survival in microbead occlusion model of ocular hypertension (Hass and Barnstable, 2019). The clinical translatability of glutamate excitotoxicity as a model of glaucomatous damage has been a topic of debate, However, Zhuang et al. (2022) recently showed that their small molecule treatment increased mitophagy and promoted RGC survival during NMDA insult.

Interestingly, Dai et al. (2018) showed an increase in Parkin, optineurin, and LC3B with IOP elevation in the LASER photocoagulation model of glaucoma. However, they also show an overall increase in less healthy mitochondria (Dai et al., 2018). The former results seem to oppose the other literature on the topic, but the latter results align with others’ observations (Coughlin et al., 2015; Dai et al., 2018; Obanina et al., 2022). These results highlight the potential pitfalls of relying on protein expression to conclude activity, the proteins involved in mitophagy may be present in higher quantity, but that does not indicate an increased activation of mitophagy. Dai et al. overexpressed Parkin in a group of mice using an AAV-2 vector, and found that during IOP elevation, Parkin overexpression led to an increased expression of Optineurin and Lamp1, an increased number of mitochondria, overall healthier mitochondria, and it protected RGCs (Dai et al., 2018). These results suggest that increasing mitophagy may be a potential therapeutic strategy for neuroprotection in glaucoma.

Some studies have found an increase in mitophagy in animal models of glaucoma. One study using MitoQC mice demonstrated increased mitophagy in mice following optic nerve crush (Rosignol et al., 2020). Being a quality control process, it is possible that mitophagy is highly elevated following the traumatic injury to the optic nerve axons and remains elevated which is highly detrimental and contributes to the rapid and drastic cell death in this model. Dixon et al. (2023) found that deletion of the Atg4b gene in DBA/2 J mice abrogated elevation of IOP and generated neuroprotective effects in RGCs and their axons. The discrepancy in the findings on mitophagy/autophagy may be related to the animal model and the duration of the pathology. Additional work is needed to provide clarification on these differences in findings about either a protective or detrimental role of mitophagy in animal models of glaucoma. Understanding the role of mitophagy in retinal ganglion cell death in glaucoma is an emerging field that could lead to new treatments to prevent vision loss in glaucoma.

## Mitophagy in age-related macular degeneration

AMD is a common ocular neurodegenerative disease in older adults with approximately 18 million patients in the United States and nearly 200 million s worldwide (Wong et al., 2014; Rein et al., 2022). The disease affects primarily the macula and is therefore characterized by progressive loss of central vision since the highest number of cone photoreceptors are present and densely packed in the macula for sharp and photopic vision. Age is a significant risk factor for AMD since the disease typically begins after age 50, and a major proportion of patients are in 60 to 90. Other risk factors for AMD are race, ethnicity, heredity, hypertension, smoking, and other environmental factors. AMD begins in the initial stages as Dry/atrophic AMD, in which there is a deposition of lipofuscin-like material called drusen. As the disease progresses, several large-sized drusen particles accumulate posterior to the RPE, disrupting the blood-retinal barrier. About 10% of Dry AMD patients progress to wet AMD, characterized by the growth of abnormal blood vessels that penetrate the Bruch’s membrane and RPE, some of which rupture and leak their contents into the subretinal space, resulting in vision loss.

Retinal pigment epithelial cells (RPE), are a supporting monolayer of epithelial cells posterior photoreceptor outer segment (POS), which forms connections with the Bruch’s membrane and plays a major role in maintaining cell homeostasis by scavenging oxygen free radicals, enabling photoreceptor renewal by phagocytosis of POS, which depend on choriocapillaris function and a supply of nutrients (McLeod et al., 2009; Zhang et al., 2023). Age and other risk factors activated by environmental exposure can accumulate reactive oxygen species, triggering cellular injury in RPE cells and induce autophagy. These conditions can cause dysfunction/degeneration of RPE and drusen (protein and lipid-rich deposits) in Bruch’s membrane, ultimately leading to the photoreceptor death and central visual loss in AMD patients. RPE degeneration could lead to reduction of the choriocapillaris vascular area and more vasoconstriction in the areas devoid of RPE (McLeod et al., 2009). Since RPE cells are involved in maintaining homeostasis and metabolic support to the adjacent photoreceptor cells, they require enormous energy to accommodate their multiple roles and functions for which they depend mainly on the mitochondria. A major function of RPE is the disposal of spent photoreceptor outer segment discs (nearly 10% of the discs are shed daily), which is mediated by an elaborate collection of hydrolytic enzymes in the lysosomes of the RPE. The accumulation of the dysfunctional/damaged mitochondria as well as the impaired phagocytosis of the dysfunctional RPE in AMD, leads to increased oxidative stress and activation of inflammatory mediators (Hollyfield et al., 2008; Gemenetzi and Lotery, 2016; Zhang et al., 2023).

Corroborative findings from several groups have shown that mitochondrial dysfunction and increased oxidative stress are the major causes of atrophic AMD (dry AMD), a subset of AMD. Studies by Feher et al. (2006) using the electron microscopic and morphometry analysis, found that compared to age-matched control subjects, AMD patients showed a significant decrease in the mitochondrial numbers and area of the mitochondria as well as a reduction in cristae with increasing age, which was accompanied by the proliferation of peroxisomes and accumulation of lipofuscin in human RPE cells. Proteomics data analysis using AMD human donor samples revealed that during the early stage of AMD, there is a decreased expression of chaperone proteins (HSP70, HSP75, HSP60, and αA crystallin) involved in protein folding and prevention of cellular damage from unfolded proteins. Interestingly, the authors found a decrease in the proteins involved in the import and folding of mitochondrial proteins (mtHSP75 and HSP60), which have implications for a decline in mitochondrial biogenesis (Nordgaard et al., 2006). Studies from the Ferrington lab found that mtDNA lesions occurred in all regions of the mt genome from AMD patients which was much higher than in age-matched control subjects, suggesting that there is a preferential damage to mtDNA during AMD progression (Karunadharma et al., 2010). Using long-extension PCR and mitochondrial genomic DNA analysis, equivalent damage of mitochondrial DNA was identified both in the macular and peripheral RPE (Terluk et al., 2015). Follow-up studies by Ferrington et al., identified that significant mitochondrial damage is associated with high-risk alleles of CFH (C), following genetic analysis using AMD and age-matched controls from human eye bank tissue samples (Ferrington et al., 2016).

Stenirri et al. (2012) found a mutation in mitochondrial ferritin (FtMt), an iron-sequestering protein that resulted in decreased protection from iron-mediated oxidative stress in AMD patients. Overexpression of mitochondrial ferritin in ARPE-19 cells resulted in protective effects by an initial increase of mitophagy, and antioxidant effects. However, increased Ftmt could upregulate HIF-1α in RPE which could promote VEGF secretion and choroidal neovascularization, a major cause of wet AMD (Wang et al., 2016).

Dib et al. (2015), showed that using ARPE-19 cells transfected with mitochondrial DNA intracellularly into the cytoplasm, were able to induce secretion of pro-inflammatory cytokines IL-6 and IL-8, 48 h post-transfection and also prime the NLRP3 inflammasome by increasing the levels of pro-IL-1β and pro-caspase1. Increase in the levels of IL-6 and IL-8 pro-inflammatory cytokines by mitochondrial DNA transfection into ARPE-1 occurred through the STING/NF-kB pathway, as evident by their siRNA knockout studies. This suggests that releasing mitochondrial DNA into the cytosol might trigger the expression of inflammatory cytokines. Brown et al. (2019) studied mitochondrial oxidative stress using conditional knockout of manganese superoxide dismutase in the RPE cells of albino BALB/cJ mice. The deletion of MnSOD in RPE mitochondria, an antioxidant enzyme, decreased visual function as measured by ERG under photo-oxidative conditions compared to age-matched controls. In the same study, authors also observed a reduction of TOMM20 expression levels along with COXIII/β-actin levels and found swollen mitochondria with disorganized cristae, leading to increased oxidative stress in RPE. The changes in the mitochondrial morphology and functioning lead to the alteration in RPE, leading to the degeneration of photoreceptors (Brown et al., 2019)^.^

Felszeghy et al. (2019) studied antioxidant defense mechanisms in RPE cell structure using NRF-2 (nuclear factor-erythroid-2 related factor-2) and PGC-1α (peroxisome proliferator-activated receptor gamma coactivator-1 alpha), global double knockout mice. They found that in NRF-2/PGC-1α dKO mice, there is elevation of various autophagosomal markers (p62/SQSTM1, Beclin-1 and LC3B) along with the increased levels of microglia marker, Iba1 and accumulation of oxidative stress markers (4-HNE) and endoplasmic reticulum stress markers (GRP78 and ATF4; Felszeghy et al., 2019). In addition, the authors also identified damaged mitochondria, dysmorphology of photoreceptor cells and vision loss (Felszeghy et al., 2019). In a subsequent study using NFE2L2/PGC1α double knockout (dKO) mice model, they observed upregulation of autophagy markers PINK1 and PARKIN together with damaged mitochondria, with no increase in the colocalization of lysosomal marker (LAMP2) and mitochondrial marker (ATP synthase β), suggesting an inability of the autophagosome to fuse with the lysosome (Sridevi Gurubaran et al., 2020). Another study by Zhang et al. (2020) showed that RPE isolated from AMD donor samples had downregulated AMPK/SIRT1 activity and decreased NAD+ which had an impact on reduced PGC-1α levels by increased acetylation and (inactivation of PGC-1α), a master regulator for mitochondrial biogenesis when compared to age-matched control samples. It is clear from these studies that mitochondrial damage and oxidative stress are key components contributing to the pathogenesis of AMD; hence, mitophagic mechanisms are essential to maintain cellular homeostasis.

Another study by Datta et al. (2023) demonstrated reduced levels of PINK1 in perifoveal RPE of early AMD eyes. In the same study, impairment of mitophagic flux was further confirmed by using pink1^−/−^ mice following intravitreal injections of carbonyl cyanide m-chlorophenyl hydrazone (CCCP) to induce mitophagy or following inhibition of the lysosomal protease activity by intraperitonial administration of leupeptin. In the ARPE-19 cells, following siRNA mediated knockdown of pink1, the authors found evidence of dysfunctional mitochondria that was detected by a decline in various mitochondrial parameters, including, mitochondrial respiration, maximal respiration, spare respiratory capacity and ATP production (Datta et al., 2023).

Among the environmental factors, cigarette smoke has been shown to produce damaging effect on RPE, such as sub-RPE deposits, oxidative damage, and apoptosis of RPE (Espinosa-Heidmann et al., 2006; Fujihara et al., 2008). In a study by Yang et al. (2020), using the RPE cells isolated from human donor samples, it was found that, RPE cells treated with hydroquinone (an important oxidant in cigarette smoke) displayed more necrotic cell death than apoptotic cells, decreased mitochondrial dehydrogenase, increased ROS production, and decreased mitochondrial bioenergetics and membrane potential. In the same study, they found that hydroquinone treated RPE cells co-treated with risuteganib, an integrin regulator, were protected from hydroquinone’s deleterious effects (Yang et al., 2020).

All these studies clearly show that damage to the mitochondria and a decline in mitophagy could be critical events in the etiology of AMD. Recent studies on phase 1 clinical trials using, Elamipretide, a mitochondrial-targeted drug, have shown a positive effect on visual function without adverse effects (Mettu et al., 2022). These studies indicate the potential for developing new approaches in treating dry AMD by targeting the mitochondria.

## Mitophagy and diabetic retinopathy

Diabetes mellitus is a severe health disorder that has drastically increased in incidence from 108 million in 1980 to 529 million patients in 2021 (Collaboration NCDRF, 2016; Collaborators GBDD, 2023). Diabetes retinopathy (DR), an ocular complication of diabetes, is highly prevalent due to the increased incidence of diabetes and afflicts nearly 80% of patients with type I (insulin-dependent) and type II (non-insulin dependent) diabetes mellitus. Diabetic retinopathy results from long periods of elevated blood glucose, and is characterized by the breakdown of the blood-retinal barrier (BRB), neovascularization and increased permeability of blood vessels. Diabetic retinopathy starts with mild non-proliferative diabetic retinopathy, characterized by tiny outpouching (microaneurysms) in retinal blood vessels. These microaneurysms progress to increased ballooning of the blood vessels, preventing retinal nourishment till a large proportion of blood vessels in the retina get blocked, resulting in a significant decrease in blood flow. This is followed by the progression of the disease to its severe form called proliferative diabetic retinopathy. The hypoxic conditions generated by the blocked blood vessels stimulate the development of abnormal new blood vessels that grow on the retina’s surface. These newly formed blood vessels are fragile, ruptured easily, and leak their contents into the vitreous humor, producing severe vision loss.

Current therapies for DR, including laser photocoagulation and anti-VEGF agents, significantly reduce the incidence of severe vision loss. However, existing therapies are ineffective over the long term in successfully halting visual decline, and are associated with troublesome side effects. It would be helpful to understand the sequelae of cellular events, starting from the elevation of blood glucose to the development of diabetic retinopathy to identify new targets and facilitate the development of efficacious drugs to treat diabetic retinopathy. A variety of mechanisms are thought to contribute to increased oxidative stress in diabetes, including, increased nonenzymatic glycosylation (glycation), autoxidative glycosylation, metabolic stress resulting from changes in energy metabolism, alterations in sorbitol pathway activity, changes in the level of inflammatory mediators and the status of antioxidant defense systems, and localized tissue damage resulting from hypoxia and ischemic reperfusion injury (Baynes, 1991). Diabetes affects the activity of many metabolic pathways (AGEs, PKC, polyol pathway, PP), and one consequence of activating these metabolic pathways is the generation of ROS (Kowluru, 2023). Thioredoxin Interacting Protein (TXNIP) is a protein that potentiates oxidative stress by inhibiting or limiting the bioavailability of thioredoxin (whose primary role is the reduction of oxidized cysteine residues and the cleavage of disulfide bonds). High glucose levels upregulate TXNIP and produce oxidative stress, resulting in mitochondrial dysfunction (Singh et al., 2021). Since chronic elevation of blood glucose in diabetes produces mitochondrial dysfunction and injury, mitophagy is crucial to clear the cell of damaged mitochondria and restore neuronal homeostasis. The extent and duration of elevation of blood glucose have a lasting effect, generating some kind of “metabolic memory” that perpetuates cellular injury even after restoring blood glucose to normal levels (Diabetes, 2015). This indicates the critical role of hyperglycemia in the dysregulation of metabolism and its damaging effects and points to importance of preventative strategies to avoid hyperglycemia as a protection against diabetic retinopathy.

Several findings point to mitochondrial injury and a compromise of mitophagy in animal models of diabetes. For instance, Alka et al. (2023) found in human retinal endothelial cells that high (20 mM) glucose levels downregulate mitofuscin 2 (MFN2) through increased acetylation of Mfn2, leading to increased mitochondrial fragmentation. The authors demonstrated that inhibition of acetylation of Mfn2 by sirtuin or overexpression of Mfn2 reduced mitochondrial fragmentation and enhanced the removal of damaged mitochondria (Alka et al., 2023). Along similar lines, Huang et al. (2022) found that under high glucose conditions, Sirt3 overexpression could activate mitophagy through Fox3a/PINK1 pathway. Other studies have demonstrated that sirtuin-mediated inhibition of FOXO3 could decrease mitophagy in RPE cells (Zhang et al., 2023). The discrepancy in findings about the role of Sirt3 in mitophagy could be due to different sets of genes activated in the two cell types. The effect of oxidative stress on mitophagy may depend on the cell type and the metabolic and cellular insults prevailing in different pathologies.

Apart from fusion, mitochondrial fission has also been implicated as a contributor to the pathological changes in diabetic retinopathy. Drp1 induces excessive mitochondrial fission, which could result in mitochondrial injury; hence, mitophagy plays a crucial role in clearing damaged mitochondria. In retinal endothelial cells, high glucose results in mitochondrial dysfunction via mitochondrial Dynamin-related protein 1 (Drp1)-mediated mitochondrial fission (Zhang et al., 2022). The authors found increased phosphorylation of Drp-1 in human retinal microvascular endothelial cells (RMECs) subjected to high glucose (33 mM) treatment for 3 days (Zhang et al., 2022). Another significant finding was that high glucose reduced mitophagy by inhibiting LC-3B-II formation and degradation of p62 (sequestosome 1). The authors found that treatment with Mdivi-1(inhibitor of Drp-1) could ameliorate intraretinal microvascular abnormalities, including, retinal vascular leakage, avascular capillaries and apoptosis in the streptozotocin model of diabetes in Sprague–Dawley rats (Zhang et al., 2022). The authors also found that 3-MA (autophagy inhibitor) worsened the HG-induced RMEC damage, while Rapamycin (autophagy agonist) protected RMEC from high glucose-mediated damage (Zhang et al., 2022). In cultured 661 W cells, Taki et al. (2020) found that high glucose (25 mM) treatment increased superoxide levels. Treatment with the autophagy inhibitor 3-methyl adenine decreased LC-3B2, while treatment with the autophagy activator Rapamycin increased LC-3B2 levels. The findings were also reflected in a decreased co-localization of mitotracker and lysotracker following treatment with 3-methyl adanine and increased co-localization of mitotracker and lysotracker by rapamycin treatment (Taki et al., 2020). The G-protein coupled bile acid receptor TGR5 is essential in energy metabolism, and a TGR5 agonist has been shown to reduce diabetes-induced retinal vascular leakage (Zhu et al., 2020). In a subsequent publication, Zhang et al. (2021) found that TGR5 inhibits mitochondrial fission and/or enhances mitophagy by regulating Drp1-HK2 signaling. Identifying receptors to activate mitophagy will help target them for neuroprotective therapies.

In another study, Drp1 was highly expressed in the retinal tissues of DR rats and HG-treated RECs. Drp1 knockdown attenuated HG-mediated increase of reactive oxygen species (ROS) levels and apoptosis in RECs (Wu et al., 2022). Moreover, Drp1 silencing inhibited the expression of autophagy-related proteins LC3-II/LC3-1 and Beclin-1 and reduced LC3 puncta in HG-treated RECs. The expression of mitochondrial marker Tom20 was reduced and the levels of mitophagy were increased in the HG-treated RECs, which was rescued by Drp1 silencing. Drp1 knockdown repressed LC3-II expression in HG-treated RECs, indicating that autophagy flux was inhibited (Wu et al., 2022). Many of the damaging effects of hyperglycemia on a decline in mitophagy and mitogenesis cannot be ameliorated by subsequent glycemic control.

For instance, Kowluru (2023) demonstrated that mitochondrial turnover rate and mitogenesis that declined following exposure of human retinal endothelial cells to high glucose, were not rescued by restoration to normal glucose levels. The authors also reported similar findings in streptozotocin-treated male Wistar rats followed by good glycemic control: clearance of damaged mitochondria and mitochondrial turnover was not enhanced after restoration of normal glucose levels (Kowluru, 2023). One study by Serikbaeva et al. (2022) found an increase in mitophagy in cultured primary human retinal endothelial cells maintained in high glucose (30 mM), suggesting that a protective response to high glucose levels may be one mechanism responsible for the delayed onset of diabetic retinopathy. Hombrebueno et al. (2019) reported that mitophagy declines in the late stages of diabetic retinopathy (DR) and is decoupled from mitochondrial biogenesis during the progression of the disease. Diabetic retinas from human postmortem donors and experimental mice exhibit a net loss of mitochondrial contents (measured by Cox4 immunostaining) during the early stages of the disease process. The authors used diabetic mitophagy-reporter mice (mitoQC-Ins2Akita) alongside pMitoTimer (a molecular clock to address mitochondrial age dynamics), and found that mitochondrial loss arose due to an inability of mitochondrial biogenesis to compensate for diabetes-mediated exacerbation of mitophagy. This suggests that while an increase in mitophagy may occur as an initial adaptive response to mitochondrial injury, long-term metabolic insults could overwhelm the capacity for quality control by mitophagy. It is plausible that different tissues in the body vary in their susceptibility to hyperglycemia which could account for the differences in the timeframe for manifestation of the pathological changes between the tissues.

Some studies have utilized agents to enhance mitophagy and generate cytoprotective effects. For example, oral administration of a novel saponin, Notoginsenoside R1 (NGR1; 30 mg/kg) for 12 weeks attenuated retinal vascular degeneration, prevented the reduction of retinal thickness, and improved retinal function in db/db mice (Zhou et al., 2019). NGR1 pre-treatment upregulated the level of PINK1 and Parkin, increased the LC3-II/LC3-I ratio, and downregulated the level of p62/SQSTM1 in rMC-1 cells induced by HG and in the retinas of db/db mice. NGR1 administration also enhanced the co-localization of GFP-LC3 puncta and MitoTracker in rMC-1 cells, indicative of the increased formation of autophagosomes (Zhou et al., 2019). Xie et al. (2021) found that high glucose levels reduced the expression of PINK1, Parkin, and VDAC1. The authors also found that over-expression of VDAC enhanced PINK1 expression, and reduced NLRP3 inflammasome activation in human retinal capillary endothelial cells (Xie et al., 2021). Doganlar et al. (2021) tested the antioxidant properties of melatonin in cultured retinal pigmented epithelial cells treated with high glucose. They found it to reduce the expression of a gene involved in mitochondrial fission (DRP1, hFis1, MIEF2, and MFF) as well as mitophagy (PINK, BNIP3, and NIX) and increase the expression of genes regulating mitochondrial biogenesis (PGC1α, NRF2, PPARγ; Doganlar et al., 2021).

The precise etiological mechanisms by which hyperglycemia contributes to diabetic retinopathy are not entirely understood. Based on several findings, it is clear that oxidative stress, mitochondrial dysfunction, and mitochondrial injury are essential contributors to the pathogenesis of diabetic retinopathy. Recent findings about a decline in mitophagy in cell culture studies and rodent models of diabetic retinopathy bring up possibilities of developing novel treatment strategies to promote mitochondrial protection and enhance bioenergetics.

## Methods for studying mitophagy

Several methods have been used to study mitophagy in these retinopathies. Several studies used western blots or immunohistochemistry to detect the expression levels of key proteins involved in mitophagy (Coughlin et al., 2015; Dai et al., 2018; Brown et al., 2019; Hombrebueno et al., 2019; Zeng et al., 2020; Xie et al., 2021; Obanina et al., 2022; Datta et al., 2023). The relative abundance of general mitochondrial markers like TOM20 can be used to estimate the number or density of mitochondria, while comparing that to the expression of key enzymes in oxidative phosphorylation (e.g., COX8A) can inform researchers about the health of the mitochondria (Hombrebueno et al., 2019). The expression of proteins involved in mitophagy have been used to draw conclusions on the prevalence of the process, but this technique is not without pitfalls. Increased expression of PINK1 means a buildup of damaged mitochondria earmarked for degradation, but provides only indirect insight into the speed with which the degradation process is happening. Increases in Parkin expression, especially when measuring the active form, is evidence of increased mitophagy, but does not confirm that the process proceeds to the end point and all mitochondria are degraded. An increase in LC3II indicates increased autophagy, but cannot be specifically attributed to mitophagy. One study used qPCR to look at the transcription of genes involved in mitophagy, but this approach has similar limitations (Doganlar et al., 2021). Analyzing the colocalization of key proteins using immunohistochemistry provides better insight into the phenomenon. Some studies have looked at the colocalization of antibody-detected proteins LC3II and TOM20, or of fluorescent dyes Lysotracker and Mitotracker (Hass and Barnstable, 2019; Zhou et al., 2019; Taki et al., 2020). Provided the authors understand the technical considerations for accurate assessment of intracellular colocalization, this technique has benefits over simply looking at protein expression. An increase in TOM20 and LC3II colocalization indicates more mitochondria are sequestered into autophagosomes, to be trafficked to the lysosome. However, it does not answer the question of whether the entire process of mitophagy is increased, or if the process is stalled at this step. It is hard to find evidence of the rate of mitochondrial degradation, but lysotracker and mitotracker may be better in that regard, as the dyes do not rely on protein markers in the mitochondria that are degraded in the lysosome.

The development of autophagy reporter mice has created new opportunities to understand the dynamics of mitophagy *in vivo*. Li et al. (2014) created a strain of mice, CAG-RFP-EGFP-LC3, which tags the autophagosome marker LC3II with green and red fluorescent proteins. When the autophagosome fuses with the lysosome, the acidic environment changes the conformation of EGFP, quenching the green fluorescence. The red fluorescent protein remains stable at low pH, so red fluorescence remains visible. These mice allow the assessment of autophagy more directly by how many autophagosomes fuse with lysosomes. This technique also allows for live cell imaging with minimal preparation. One limitation to studying mitophagy in that without labeling the mitochondria, it is impossible to distinguish mitophagy from general autophagy (Li et al., 2014). However, the limitation of this technique can be overcome by the use of immunostaining, dyes such as mitotracker, or further genetic manipulation to express a fluorescent protein on the mitochondria. The latter has several technical limitations that need to be overcome, for example, selection of a fluorophore that can be inserted that has minimal overlap with the other two and is stable in acidic environments may be difficult. Since the development of CAG-RFP-EGFP-LC3 mice, some groups have engineered pH-sensitive dual fluorophore systems attached to the mitochondria for the quantification of mitophagy. The first of these is the MitoQC mouse, developed by McWilliams et al. (2016), constitutively express mCherry and GFP tagged FIS1 on the outer mitochondrial membrane. When the mitochondria is trafficked to a lysosome, the acidic environment changes the conformation of GFP and the green fluorescence is quenched (McWilliams et al., 2016). This mouse model, with the addition of a mitochondrial age marker, has been used to study mitophagy in diabetic retinopathy (Hombrebueno et al., 2019). Shortly after the advent of the MitoQC mouse, the Mt-Keima mouse was developed using a single fluorophore, keima red, which fluoresces at a different wavelength in acidic environments compared to physiological pH (Sun et al., 2017). A recent paper suggests that Mt-Keima mice are a more sensitive detector of parkin-dependent mitophagy than MitoQC, which they attribute to the fluorescent probe in MitoQC localized on the outer mitochondrial membrane where it may be subject to proteolytic cleavage outside of the lysosome (Liu et al., 2021). Mt-Keima mice use a Cox VIII presequence for mitochondrial targeting of their fluorescent probe, which has been used to target proteins to the mitochondrial matrix (Chinnery et al., 1999; Katayama et al., 2011). More recently, Katayama et al. (2020) have created a new mitophagy reporter mouse model they dub Mito-SRAI. This new mouse model is claimed to yield more reproducible, easier to interpret, results (Katayama et al., 2020). This is due in part to the use of fluorescent resonance energy transfer (FRET), fluorophores were carefully selected such that, when the fluorophores are paired, activation of the donor fluorophore causes a highly efficient energy transfer to the acceptor fluorophore, producing a large emission from the acceptor fluorophore, this effect diminishes exponentially as the distance between the fluorophores increases. The authors state the ease of detection makes this probe ideal for use in high throughput assays, which may aid in the discovery of drugs to treat ocular diseases by modulating mitophagy.

While the advent of *in-vivo* fluorescent probes has revolutionized the study of mitophagy, the gold standard for visualizing mitophagy continues to be transmission electron microscopy (TEM). TEM allows for the direct visualization of subcellular structures, but it has several disadvantages. TEM is expensive, the preparations are time consuming, and requires great skill. Unlike other tissues where a small biopsy can be sent for TEM, the best results require the fixation of the whole eye, leaving no sample left for other assays (Hayden and Meseck, 2021). The contralateral eye is still available for other assays, but it may be difficult to get samples for all experiments. Upon dissection of the contralateral eye, it is possible to bisect it midsagittal, fix half the eye for paraffin-embedded sectioning, and isolate retinal ganglion cells from the other half for protein extraction, but the retina is more easily detached from half the eye cup, leading to problems embedding and sectioning, and the amount of protein extracted is severely limited as well. With these limitations, groups who do electron microscopy typically look at the optic nerve (Coughlin et al., 2015; Dai et al., 2018). However, mitophagy levels in the optic nerve axon may not be the same as the RGC soma, because there is some evidence that mitochondria in the axons are exported to astrocytes for degradation rather than processed within the axon (Davis et al., 2014). This may explain why previous studies using TEM of the optic nerve have not observed changes in mitophagy, but they do observe an increase in malformed mitochondria, which could be transported to the optic nerve because they are not being degraded in the RGC soma (Coughlin et al., 2015; Dai et al., 2018). As technology continues to develop, new approaches and techniques will be developed to overcome current limitations in these approaches.

## Conclusion

The precise role of mitophagy in ocular neurodegeneration is still being actively investigated; however, an abundance of data suggests that a decline in mitophagy could be a contributor to neurodegenerative effects. There are also findings that suggest an increase in mitophagy in some animal models of ocular neurodegeneration. Most studies have used immunohistochemical labeling for various markers of mitochondria, autophagosomes, and lysosomes to make conclusion about mitophagic flux. There is a need for more dynamic assays to figure out the spatiotemporal changes in mitophagic flux in various pathologies. The key steps responsible for oxidative stress-mediated damage of mitochondria and the cellular events leading to a decline in mitophagy are not entirely understood. Unraveling these pathways will facilitate an in-depth understanding of the mitophagic mechanisms and help develop therapeutic targets to quell neurodegeneration in various neurodegenerative disorders.

## Author contributions

CB: Conceptualization, Visualization, Writing – original draft, Writing – review & editing. BK: Conceptualization, Validation, Writing – original draft, Writing – review & editing. DS: Conceptualization, Funding acquisition, Validation, Writing – original draft, Writing – review & editing. RK: Conceptualization, Funding acquisition, Supervision, Validation, Writing – original draft, Writing – review & editing.
